# Supplementary material for: Typical thermalization of low-entanglement states
Source: Commun Phys. 2025 Jul 17;8(1):301. doi: 10.1038/s42005-025-02161-7 (PMC12270909; doi:10.1038/s42005-025-02161-7)
Supplement: Supplementary file 2 — Supplemental material [file 42005_2025_2161_MOESM2_ESM.pdf]

Supplementary Information: Typical thermalization of low-entanglement states

Christian Bertoni,[1] Clara Wassner,[1] Giacomo Guarnieri,[2,1] and Jens Eisert[1,3]
[1]*Dahlem Center for Complex Quantum Systems, Freie Universität, 14195 Berlin, Germany*
[2]*Department of Physics, University of Pavia, Via Bassi 6, 27100, Pavia, Italy*
[3]*Helmholtz Center Berlin, 14109 Berlin, Germany*
(Dated: May 7, 2025)

I. SUPPLEMENTARY NOTE 1: EQUIVALENCE OF ENSEMBLES

The main purpose of this section is to prove Theorem 1 of the main text in a more general version. For what follows, we will need two auxiliary lemmas, both stated and proven in Ref. [1], the final result will follow from a similar argument to the one used to prove equivalence of statistical mechanics ensembles in the same work. The first one states that a state close in relative entropy to some other state with exponential decay of correlations will also be locally indistinguishable from that state.

Lemma 1 ([1], Proposition 2)**.** *Let ρ be a state with correlation length ξ and let τ be a state. Let $\mathcal{C}_l$ be the set of hypercubes in Λ with edge length l, if for $\epsilon > 0$*

$$S(\tau||\rho) + 3 + \epsilon^{\frac{D+1}{D+2}} \frac{2\xi \ln(d) l^D + l + 2}{\xi \ln(2)} + \epsilon^{\frac{D+1}{D+2}} \log(N) \leq \epsilon \left(\frac{N}{\ln(4)^D \xi^D} \right)^{\frac{1}{D+1}} \tag{1}$$

then

$$D_l(\tau, \rho) \leq 7\sqrt{\epsilon^{\frac{D+1}{D+2}}}. \tag{2}$$

This theorem has important consequences. In less formal terms, it implies that bounding the relative entropy between the two states bounds their local distinguishability, assuming some bounds on ϵ and the local regions:

Corollary 1 (Local indistinguishability from relative entropy closeness)**.** *Let ρ be a state with correlation length ξ and let τ be a state. Suppose that for some ϵ, we have*

$$S(\tau||\rho) \leq \epsilon N^{\frac{1}{D+1}}. \tag{3}$$

Let

$$\tilde{\epsilon} := \frac{4}{(\xi \ln(4))^{\frac{D}{D+1}}} \epsilon. \tag{4}$$

If

$$\tilde{\epsilon} \geq \left(\frac{\ln(4)^D \xi^D}{N} \right)^{\frac{D+2}{D+1}} (4 \log(N))^{D+2} \tag{5}$$

then for

$$l^D \leq \frac{1}{4} \frac{\xi \ln(2)}{2\xi \ln(d) + 3} \tilde{\epsilon}^{\frac{1}{D+2}} \left(\frac{N}{\xi^D \ln(4)^D} \right)^{\frac{1}{D+1}} \tag{6}$$

we have

$$D_l(\tau, \rho) \leq 7\sqrt{\tilde{\epsilon}^{\frac{D+1}{D+2}}}. \tag{7}$$

The second lemma is a variant of the Berry-Esseen theorem for quantum lattice systems. We denote with $\{|\nu\rangle\}_\nu$ and $\{E_\nu\}_\nu$ the eigenstates and corresponding eigenvalues of a local Hamiltonian H. If a state ρ is uncorrelated, the energy probability distribution $\langle \nu|\rho|\nu\rangle$ behaves like a sum of independent random variables, and converges to a normal distribution with the system size. The Berry-Esseen theorem for quantum lattice systems gives system-size dependent bounds on the speed of convergence of the distribution.

Definition 1 (Berry-Esseen error). *Let ρ be a state and H a local Hamiltonian, define $\mu := \mathrm{tr}\,(\rho H)$ and $\sigma^2 = \mathrm{tr}\,\left(\rho(H-\mu)^2\right)$ and let the functions F and G be defined as*

$$F(x) = \sum_{\nu: E_\nu \leq x} \langle \nu|\rho|\nu\rangle, \qquad G(x) = \frac{1}{\sqrt{2\pi\sigma^2}} \int_{-\infty}^{x} \mathrm{e}^{-\frac{(y-x)^2}{2\sigma^2}}, \tag{8}$$

then the Berry-Esseen error is the supremum of the difference of these two cumulative distribution functions

$$\zeta_N(\rho, H) = \sup_{x\in\mathbb{R}} |F(x) - G(x)|. \tag{9}$$

Lemma 2 ([1], Lemma 8). *Suppose $g_\beta(H) = \frac{\mathrm{e}^{-\beta H}}{Z(\beta)}$ has exponential decay of correlations, let $\sigma^2 = \mathrm{tr}\,\left(g_\beta(H)H^2\right) - \mathrm{tr}\,\left(g_\beta(H)H\right)^2$ and assume $\sigma^2 \geq \Omega(N)$. Then*

$$\zeta_N(g_\beta(H), H) \leq R\,\frac{\ln(N)^{2D}}{\sigma} \tag{10}$$

where R is a constant depending on the correlation length, the locality of the Hamiltonian, and the dimension of the lattice.

A tighter bound, without the logarithmic factor, holds for Gibbs states at sufficiently high temperature [2]. Throughout the document, we define $\zeta_N := \zeta_N(g_\beta(H), H)$. We are now ready to prove the following statement. This is a more general version of the main theorem of [1] for the case of the GmE and the proof follows from appropriately adapting the argument in that work.

Theorem 1 (Thermality condition). *Let H be a local Hamiltonian and let*

$$\begin{aligned} &\frac{1}{6\sqrt{2\pi}}\sigma N^{\frac{-1}{D+1}} \geq \epsilon \geq \max\left\{\log\left(\frac{1}{\tilde{\zeta}_N}\right), \mathrm{e}^{1+\sqrt{5}}\ln(N)^{2D}\right\} N^{\frac{-1}{D+1}}, \\ &3\sqrt{2\pi}\,\mathrm{e}^2\sigma\tilde{\zeta}_N \leq \Delta \leq \sigma\sqrt{\ln\left(\frac{\epsilon N^{\frac{1}{D+1}}}{R\ln(N)^{2D}}\right)}, \end{aligned} \tag{11}$$

for some $\tilde{\zeta}_N \geq \zeta_N$ such that $\sigma\tilde{\zeta}_N \leq R\ln(N)^{2D}$. Let τ be a state supported only on eigenstates with energy in $I = [E+\Delta, E-\Delta]$ for some energy E. Divide the interval I into subintervals $[e_k, e_{k+1})$ and suppose $\delta_k = |e_{k+1} - e_k|$ satisfies

$$\delta_k^* := 3\sqrt{2\pi}\,\mathrm{e}^{\frac{1}{2}\left(\frac{\Delta(k)}{\sigma}+2\right)^2}\sigma\tilde{\zeta}_N \leq \delta_k \leq \sigma \tag{12}$$

where we have defined

$$\Delta(k) := \begin{cases} |e_k - E| & \text{if } E < e_k, \\ |e_{k+1} - E| & \text{if } E > e_{k+1}, \\ 0 & \text{if } E \in [e_k, e_{k+1}]. \end{cases} \tag{13}$$

Call the projector onto the eigenstates contained in the k-th window Π_k. If for each k

$$S(\tau) \geq \sum_k \mathrm{tr}\,(\Pi_k \tau)\log\left(\mathrm{tr}\,(\Pi_k)\right) - \epsilon N^{\frac{1}{D+1}}, \tag{14}$$

then for any l such that

$$l^D \leq \frac{1}{4}\left(\frac{4}{(\xi\ln(4))^{\frac{D}{D+1}}}\right)^{\frac{1}{D+2}} \frac{\xi\ln(2)}{2\xi\ln(d)+3}\epsilon^{\frac{1}{D+2}} \left(\frac{N}{\xi^D\ln(4)^D}\right)^{\frac{1}{D+1}}, \tag{15}$$

we have

$$D_l(\rho, g_\beta(H)) \leq 7\left(\frac{4\left(\beta\log(\mathrm{e})3\sqrt{2\pi}+1\right)}{(\xi\ln(4))^{\frac{D}{D+1}}}\right)^{\frac{1}{2}\frac{D+1}{D+2}} \epsilon^{\frac{1}{2}\frac{D+1}{D+2}} \tag{16}$$

for any β such that $|E - \mathrm{tr}\,(Hg_\beta(H))| \leq \sigma$ and $g_\beta(H)$ has exponential decay of correlations.

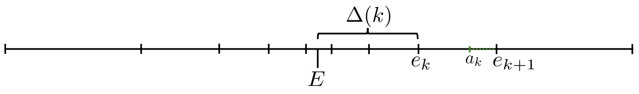

Supplementary Figure S1. A sketch of the interval Δ and the sub-intervals δ_k appearing in Theorem 1.

Proof. We would like to apply Lemma 1. We then need to bound the relative entropy $S(\tau||g_\beta(H))$. We begin by writing, by definition of relative entropy (here $\log$ denotes the logarithm in base 2),

$$S(\tau||g_\beta(H)) = -S(\tau) + \beta\log(\mathrm{e})\mathrm{tr}\,(H\tau) + \log(Z(\beta)). \tag{17}$$

In a next step, we have

$$\mathrm{tr}\,(H\tau) = \sum_k \mathrm{tr}\,(\Pi_k H \Pi_k \tau) \leq \sum_k \mathrm{tr}\,(\tau_k)\, e_{k+1}, \tag{18}$$

where we have defined $\tau_k := \Pi_k \tau \Pi_k$ and have used that $\Pi_k H \Pi_k \leq e_{k+1}$. We can then conclude that

$$S(\tau||g_\beta(H)) \leq -S(\tau) + \sum_k \mathrm{tr}\,(\tau_k)\,(\log(\mathrm{e})\beta e_{k+1} + \log(Z(\beta))). \tag{19}$$

We define

$$Z_k(\beta) := \sum_{E_\nu \in [a_k, e_{k+1}]} \mathrm{e}^{-\beta E_\nu} \tag{20}$$

for some $a_k \geq e_k$ to be defined later. Then we write for each k,

$$Z(\beta) = \frac{Z(\beta)}{Z_k(\beta)} Z_k(\beta). \tag{21}$$

At this point, notice that

$$Z_k(\beta) = \sum_{E_\nu \in [a_k, e_{k+1}]} \mathrm{e}^{-\beta E_\nu} \leq d_k \mathrm{e}^{-\beta a_k}, \tag{22}$$

where we have defined $d_k := \mathrm{tr}\,(\Pi_k)$. We then have

$$S(\tau||g_\beta(H)) \leq -S(\tau) + \sum_k \mathrm{tr}\,(\tau_k) \log(d_k) + \sum_k \mathrm{tr}\,(\tau_k) \log\left(\frac{Z(\beta)}{Z_k(\beta)}\right) + \beta\log(\mathrm{e}) \sum_k \mathrm{tr}\,(\tau_k)\,\tilde{\delta}_k, \tag{23}$$

with $\tilde{\delta}_k := e_{k+1} - a_k$. We now need to show that $Z(\beta)/Z_k(\beta)$ is not too large for an appropriate choice of intervals. To this end, we write

$$\frac{Z_k(\beta)}{Z(\beta)} = \sum_{E_\nu \in [a_k, e_{k+1}]} \langle \nu | g_\beta(H) | \nu \rangle. \tag{24}$$

We proceed by using that the energy distribution of a thermal state is close to a Gaussian (Lemma 2). Define the functions F and G as

$$F(x) := \sum_{E_\nu \leq x} \langle \nu | g_\beta(H) | \nu \rangle, \qquad G(x) = \frac{1}{\sqrt{2\pi\sigma^2}} \int_{-\infty}^{x} \mathrm{d}x\, \mathrm{e}^{-\frac{(x-E_\beta)^2}{2\sigma^2}} \tag{25}$$

with $E_\beta := \mathrm{tr}\,(H g_\beta(H))$. Then

$$\begin{aligned} \frac{Z_k(\beta)}{Z(\beta)} &= F(e_{k+1}) - F(a_k) \geq G(e_{k+1}) - G(a_k) - 2\mathrm{sup}|F(x) - G(x)| \\ &\geq G(e_{k+1}) - G(a_k) - 2\zeta_N. \end{aligned} \tag{26}$$

Recall that we wish to lower bound $Z_k(\beta)/Z(\beta)$. If $\tilde{\delta}_k$ is too small, $G(e_{k+1}) - G(a_k)$ will also be small and the lower bound in the above equation will be negative, hence we need to pick $\tilde{\delta}_k$ large enough such that $G(e_{k+1}) - G(a_k) \geq 3\tilde{\zeta}_N$ for some $\tilde{\zeta}_N \geq \zeta_N$ such that $\sigma\tilde{\zeta}_N \leq R\ln(N)^{2D}$, which will yield $Z_k(\beta)/Z(\beta) \geq \tilde{\zeta}_N$. The reason why we introduce $\tilde{\zeta}_N$ is that if ζ_N itself is too small, the lower bound $Z_k(\beta)/Z(\beta) \geq \zeta_N$ might be too loose later. We have

$$G(e_{k+1}) - G(a_k) = \frac{1}{\sqrt{2\pi\sigma^2}} \int_{a_k}^{e_{k+1}} \mathrm{d}x\, \mathrm{e}^{-\frac{(x-E_\beta)^2}{2\sigma^2}} \geq \tilde{\delta}_k \frac{1}{\sqrt{2\pi\sigma^2}} \exp\left(- \max_{x\in[a_k,e_{k+1}]} \frac{(x-E_\beta)^2}{2\sigma^2}\right). \tag{27}$$

Hence, in order for $G(e_{k+1}) - G(a_k) \geq 3\tilde{\zeta}_N$ we need to pick a_k such that

$$\tilde{\delta}_k \geq 3\sqrt{2\pi}\sigma\tilde{\zeta}_N \exp\left(\max_{x\in[a_k,e_{k+1}]} \frac{(x-E_\beta)^2}{2\sigma^2}\right). \tag{28}$$

We have

$$\frac{|x-E_\beta|}{\sqrt{2}\sigma} \leq \frac{|x-E| + |E-E_\beta|}{\sqrt{2}\sigma} \leq \frac{1}{\sqrt{2}}\left(2 + \frac{\Delta(k)}{\sigma}\right) \tag{29}$$

where we have used that by assumption $|E - E_\beta| \leq \sigma$ and that since $x \in [a_k, e_{k+1}]$ we have

$$|x - E| \leq \Delta(k) + \delta_k \leq \Delta(k) + \sigma, \tag{30}$$

where we have imposed $\delta_k \leq \sigma$. We set

$$\tilde{\delta}_k = \delta_k^* := 3\sqrt{2\pi}\sigma\tilde{\zeta}_N \exp\left(\frac{1}{2}\left(\frac{\Delta(k)}{\sigma} + 2\right)^2\right). \tag{31}$$

We have $G(e_{k+1}) - G(a_k) \geq 3\tilde{\zeta}_N$, and naturally, since $\tilde{\delta}_k \leq \delta_k$, in order to be able to choose $\tilde{\delta}_k = \delta_k^*$, we need $\delta_k \geq \delta_k^*$. Hence, if

$$\delta_k^* \leq \delta_k \leq \sigma, \tag{32}$$

we have

$$\frac{Z(\beta)}{Z_k(\beta)} \leq \frac{1}{\tilde{\zeta}_N}. \tag{33}$$

We need to ensure that the ranges for δ_k exist. Notice that since by definition $\Delta(k) \leq \Delta$

$$\frac{\Delta(k)}{\sigma} + 2 \leq \sqrt{\ln\left(\frac{\epsilon N^{\frac{1}{D+1}}}{R\ln(N)^{2D}}\right)} + 2 \leq \sqrt{2\ln\left(\frac{\epsilon N^{\frac{1}{D+1}}}{R\ln(N)^{2D}}\right)}, \tag{34}$$

where we for the last inequality we have used

$$\epsilon \geq \mathrm{e}^{1+\sqrt{5}} \ln(N)^{2D} N^{\frac{-1}{D+1}}. \tag{35}$$

Hence,

$$\delta_k^* \leq 3\sqrt{2\pi}\epsilon N^{\frac{1}{D+1}} \frac{\sigma\tilde{\zeta}_N}{R\ln(N)^{2D}} \leq \frac{1}{2}\sigma \tag{36}$$

where we have used the assumed upper bound on ϵ. Hence, if for some k we have $\Delta(k) + \delta_k \geq \Delta$, that is, the lower bound on the next interval requires to leave I, we can simply redefine δ_{k-1} to extend all the way to the edge of I. We can now go back to equation Eq. (23) and we have

$$S(\tau||g_\beta(H)) \leq -S(\tau) + \sum_k \mathrm{tr}\,(\tau_k)\log(d_k) + \beta\log(\mathrm{e})3\sqrt{2\pi}\sigma\tilde{\zeta}_N\epsilon N^{\frac{1}{D+1}} + \log\left(\frac{1}{\tilde{\zeta}_N}\right). \tag{37}$$

Furthermore, we use

$$\epsilon \geq \log\left(\frac{1}{\tilde{\zeta}_N}\right) N^{\frac{-1}{D+1}} \tag{38}$$

which ensures that $\log\left(1/\tilde{\zeta}_N\right) \leq \epsilon N^{\frac{1}{D+1}}$, hence overall,

$$S(\tau||g_\beta(H)) \leq -S(\tau) + \sum_k \mathrm{tr}\left(\tau_k\right)\log(d_k) + \left(\beta\log(\mathrm{e})3\sqrt{2\pi}\sigma\tilde{\zeta}_N + 1\right)\epsilon N^{\frac{1}{D+1}}. \tag{39}$$

If we impose

$$\sum_k \mathrm{tr}\left(\tau_k\right)\log(d_k) - S(\tau) \leq \epsilon N^{\frac{1}{D+1}}, \tag{40}$$

we have that

$$S(\tau||g_\beta(H)) \leq (\beta\log(\mathrm{e})3\sqrt{2\pi}\sigma\tilde{\zeta}_N + 2)\epsilon N^{\frac{1}{D+1}} \leq (\beta\log(\mathrm{e})3\sqrt{2\pi} + 2)\epsilon N^{\frac{1}{D+1}}, \tag{41}$$

where we have used $\sigma\tilde{\zeta}_N \leq R\ln(N)^{2D}$. The result then follows from Corollary 1. □

Theorem 1 and its approximate version (Eq. (11) in the main text) follow from the following more general statement.

Theorem 2 (Ensemble equivalence)**.** *Let H be a local Hamiltonian and β be an inverse temperature for which the Gibbs state $g_\beta(H)$ has exponential decay of correlations and standard deviation $\sigma \geq \Omega(\sqrt{N})$. Suppose $\sigma\zeta_N \leq R\ln(N)^{2D}N^{-\kappa}$ for $R, \kappa \geq 0$ constant. Let ω denote an approximate GmE state with Δ, δ, η satisfying*

$$\begin{aligned} &\mathrm{e}^{\Delta^2/\sigma^2} \leq \frac{N^{\frac{1-\alpha}{D+1}}}{R\ln(N)^{2D}}, \\ &3\sqrt{2\pi}N^{\frac{1-\alpha}{D+1}-\kappa} \leq \delta \leq \sigma, \\ &\eta \leq N^{\frac{1-\alpha}{D+1}} \end{aligned} \tag{42}$$

with $\alpha \in [0,1)$, such that $|E - E_\beta| \leq \sigma$. Then for any side length l such that $l^D \leq C_1 N^{\frac{1}{D+1}-\gamma_1\alpha}$, the following holds

$$D_l(\omega, g_\beta(H)) \leq C_2 N^{-\gamma_2\alpha} + 2(1-p_\Delta), \tag{43}$$

with C_1, C_2 being system-size independent constants, and γ_1, γ_2 only depend on the dimension of the lattice D.

Proof. Choose

$$\epsilon := N^{-\frac{\alpha}{D+1}} \tag{44}$$

with $\alpha < 1$ constant and set $\tilde{\zeta}_N := R\ln(N)^{2D}N^{-\kappa}/\sigma \geq \zeta_N$. Then we have

$$\Delta \leq \sigma\sqrt{\ln\left(\frac{N^{\frac{1-\alpha}{D+1}}}{R\ln(N)^{2D}}\right)} = \sigma\sqrt{\ln\left(\frac{\epsilon N^{\frac{1}{D+1}}}{R\ln(N)^{2D}}\right)}. \tag{45}$$

We would like to apply Theorem 1. For this, we set $\delta_k := \delta$, hence we need $\delta \geq \delta_k^*$ for all k. As shown in the proof of Theorem 1, for N sufficiently large we have by using $\Delta(k) \leq \Delta$

$$\delta_k^* \leq 3\sqrt{2\pi}\epsilon N^{\frac{1}{D+1}}\frac{\sigma\tilde{\zeta}_N}{R\ln(N)^{2D}} = 3\sqrt{2\pi}N^{\frac{1-\alpha}{D+1}-\kappa} \tag{46}$$

Then, for all k, choose

$$\delta = 3\sqrt{2\pi}N^{\frac{1-\alpha}{D+1}-\kappa}. \tag{47}$$

The subdivision then satisfies the assumptions of Theorem 1 for the chosen ϵ. Let $\tau = \sum_{k=1}^K q_k\tilde{\omega}_{\delta_k}$ such that $\omega = p_\Delta\tau + (1 - p_\Delta)\rho_{\mathrm{tail}}$. We have

$$\|\omega - \tau\|_1 = \|\omega - p_\Delta\tau + p_\Delta\tau - \tau\|_1 \leq 2(1-p_\Delta). \tag{48}$$

We then have

$$D_l(\omega, g_\beta(H)) \leq D_l(\tau, g_\beta(H)) + 2(1 - p_\Delta). \tag{49}$$

Furthermore, by the concavity of the Von Neumann entropy

$$S(\tau) \geq \sum_k q_k S(\tilde{\omega}_{\delta_k}) \geq \sum_k q_k S(\omega_{\delta_k}) - N^{\frac{1-\alpha}{D+1}}. \tag{50}$$

Notice that $\mathrm{tr}\,(\Pi_k \tau) = q_k$ and $S(\omega_{\delta_k}) = \log(d_k)$. By Theorem 1,

$$D_l(\tau, g_\beta(H)) \leq C_2 N^{-\gamma_2 \alpha}, \tag{51}$$

for C_2, γ_2 constants. □

Theorem 1 in the main text is the special case where $p_\Delta = 1$ and $\tilde{\omega}_{\delta_k} = \omega_{\delta_k}$.

II. SUPPLEMENTARY NOTE 2: WEINGARTEN CALCULUS

In what follows, we will often need to compute averages of random unitaries. Here we very briefly give the necessary basics and notation. For a comprehensive introduction, see Ref. [3]. Let $\mathcal{U}(d)$ be the unitary group of dimension d and $\mu_H(d)$ be the Haar measure thereon. We will be interested in computing averages involving unitaries of the form $U^{\otimes k}$, for some integer k, where U is drawn from $\mu_H(d)$. In reality, U needs only to be drawn from a unitary k-design. In particular, we have, for any operator X acting on $\mathbb{C}^{d^{\otimes k}}$,

$$\mathbb{E}_{U \sim \mu_H(d)} \left(U^{\otimes k} X U^{\otimes k^\dagger} \right) = \sum_{\sigma, \tau \in S_k} \mathrm{Wg}(\sigma\tau^{-1}, d) \mathrm{tr}\,(R_\sigma X)\, R_\tau \tag{52}$$

where S_k is the symmetric group of k elements with R being a representation such that for $\sigma \in S_k$, R_σ permutes the tensor spaces

$$R_\sigma |i_1, \ldots, i_k\rangle = |i_{\sigma(1)}, \ldots, i_{\sigma(k)}\rangle \tag{53}$$

in $\mathbb{C}^{d^{\otimes k}}$ according to σ. $\mathrm{Wg}(\sigma\tau^{-1}, d)$ is the Weingarten function. Table S1 and Table S2 give its values for $k = 2$ and $k = 4$, respectively. Such tables can be generated using the software developed in Ref. [4] and tables up to $k = 20$ are available at the corresponding GitHub repository.

Cycle type of σ	$\mathrm{Wg}(\sigma, d)$
$(1, 1)$	$\frac{1}{d^2-1}$
(2)	$\frac{1}{-d(d^2-1)}$

Supplementary Table S1. Weingarten functions for $k = 2$.

III. SUPPLEMENTARY NOTE 3: THERMALIZATION UNDER TYPICAL HAMILTONIANS

We now apply our thermality condition to prove thermalization with high probability of appropriate states under a suitable slightly perturbed Hamiltonian. Before proving the theorems in the main text, we discuss tails decay for states with exponential decay of correlations. The following has been proven in Ref. [5].

Theorem 3 (Spectral tail bound)**.** *Let ρ be a state and H be a local Hamiltonian. Let Π_Δ be the projector onto all eigenstates of H with energy E such that $|E - \mathrm{tr}\,(\rho H)| \leq \Delta$. Then,*

1. if ρ is a product state, and $\Delta \geq g_0 \sqrt{N}$

$$\mathrm{tr}\,((1 - \Pi_\Delta)\rho) \leq \mathrm{e}^{-s_0 \frac{\Delta^2}{N}}, \tag{54}$$

where g_0, s_0 are system size independent constants.

Cycle type of σ	$\mathrm{Wg}(\sigma, d)$
$(1,1,1,1)$	$\frac{d^4-8d^2+6}{d^8-14d^6+49d^4-36d^2}$
$(2,1,1)$	$\frac{1}{d^5-10d^3+9d}$
$(3,1)$	$\frac{2d^2-3}{d^8-14d^6+49d^4-36d^2}$
$(2,2)$	$\frac{d^2+6}{d^8-14d^6+49d^4-36d^2}$
(4)	$-\frac{1}{d^7-14d^5+49d^3-36d}$

Supplementary Table S2. Weingarten functions for $k = 4$.

2. *if ρ has exponential decay of correlations, and $\Delta \geq g_\xi \sqrt{N}$*

$$\mathrm{tr}\left((1-\Pi_\Delta)\rho\right) \leq \mathrm{e}^{-s_\xi \left(\frac{\Delta^2}{N}\right)^{\frac{1}{D+1}}}, \tag{55}$$

where g_ξ, s_ξ are system size independent constants depending on the correlation length ξ.

We repeat here the definition of the ensemble $\mathcal{E}(\delta)$: we divide the energy spectrum into intervals $\{I_k\}_{k=1}^K$ of equal energy width δ, the eigenstates in the k-th window span a vector space which we call $\mathcal{W}_k$. $\mathcal{E}(\delta)$ is then the ensemble of random unitaries of the form

$$U = \bigoplus_{k=1}^K U_k, \tag{56}$$

where U_k is drawn from the Haar measure on the unitary group acting on $\mathcal{W}_k$ independently for every k. Generally, we will denote by Π_k the projector onto $\mathcal{W}_k$, by W_k the set of indices ν such that $E_\nu \in I_k$, and by $d_k := |W_k|$ the number of eigenstates contained in each window. In addition we will denote $\rho_k = \Pi_k \rho \Pi_k$. For convenience, let us prove a fact that will be useful later.

Lemma 3 (Perturbing Hamiltonians)**.** *Let U be drawn from $\mathcal{E}(\delta)$. Then*

$$\|H - UHU^\dagger\|_\infty \leq \delta. \tag{57}$$

Proof. Since both H and U commute with Π_k, we have

$$(UHU^\dagger - H)^2 = \sum_{k=1}^K \Pi_k (UHU^\dagger - H)^2 \Pi_k = \sum_{k=1}^K (U_k H_k U_k^\dagger - H_k)^2 \tag{58}$$

with $H_k := \Pi_k H \Pi_k$. Let ρ be a state and define $\rho_k := \Pi_k \rho \Pi_k$. We then have

$$\mathrm{tr}\left(\rho(UHU^\dagger - H)^2\right) = \sum_{k=1}^K \mathrm{tr}\left(\rho_k (U_k H_k U_k^\dagger - H_k)^2\right) \leq \sum_{k=1}^K \mathrm{tr}\left(\rho_k\right) \|U_k H_k U_k^\dagger - H_k\|_\infty^2, \tag{59}$$

where we have used Hölder's inequality and the fact that since $\rho_k > 0$, $\|\rho_k\|_1 = \mathrm{tr}\left(\rho_k\right)$. Define

$$m_k := e_k + \frac{\delta}{2} = \frac{e_k + e_{k+1}}{2}, \tag{60}$$

then

$$\|U_k H_k U_k^\dagger - H_k\|_\infty = \|U_k H_k U_k^\dagger - m_k \Pi_k + m_k \Pi_k - H_k\|_\infty \leq 2\|H_k - m_k \Pi_k\|_\infty \leq \delta^2. \tag{61}$$

Hence,

$$\mathrm{tr}\left(\rho(UHU^\dagger - H)^2\right) \leq \delta^2, \tag{62}$$

which shows $\|UHU^\dagger - H\|_\infty \leq \delta$ by the definition of operator norm. □

First, we prove Lemma 1 in the main text in the more general case where eigenvalues can be degenerate.

Lemma 4 (Approximate GmE at equilibrium)**.** *Let ρ be a product state and H be a local Hamiltonian. Let*

$$\rho_\infty^{UHU^\dagger} = \lim_{T\to\infty}\frac{1}{T}\int_0^T \mathrm{e}^{-iUHU^\dagger t}\rho \mathrm{e}^{iUHU^\dagger t} \tag{63}$$

where U is drawn from $\mathcal{E}(\delta)$. Consider the interval $I = [E-\Delta, E+\Delta]$ around $E = \mathrm{tr}\,(\rho H)$ with $\Delta \geq \omega(\sqrt{N})$ an integer multiple of δ, then

$$\rho_\infty^{UHU^\dagger} = p_\Delta\left(\sum_{k:I_k\subset I} q_k\tilde{\omega}_{\delta_k}\right) + (1-p_\Delta)\rho_{\mathrm{tail}} \tag{64}$$

with $p_\Delta \geq 1-\mathrm{e}^{-c_1\frac{\Delta^2}{N}}$ and $\sum_{k:I_k\subset I} q_k = 1$. Moreover, with probability at least $1-(\mathcal{D}+1)2^{-r}$, where $\mathcal{D}$ is the degeneracy of the most degenerate eigenvalue with energy inside I, we have

$$\sum_{k:I_k\subset I} q_k(S(\omega_{\delta_k}) - S(\tilde{\omega}_{\delta_k})) \leq r, \tag{65}$$

where c_1 is a system-size independent constant.

Proof. Remember that we denote the spectral decomposition of H as $H = \sum_\nu E_\nu P_\nu$. We have

$$\rho_\infty^{UHU^\dagger} = \sum_\nu UP_\nu U^\dagger \rho U P_\nu U^\dagger. \tag{66}$$

Notice that if $E_\nu \in I_k$, then P_ν is a projector in $\mathcal{W}_k$, and since the unitaries U preserve the spaces $\mathcal{W}_k$, $UP_\nu U^\dagger$ is also a projector in $\mathcal{W}_k$. Let W_k be the set of indices ν such that $E_\nu \in I_k$. Then

$$\rho_\infty^{UHU^\dagger} = \sum_{k:I_k\subset I}\sum_{\nu\in W_k} U_kP_\nu U_k^\dagger\rho_k U_k P_\nu U_k^\dagger + \sum_{k:I_k\not\subset I}\sum_{\nu\in W_k} U_kP_\nu U_k^\dagger\rho_k U_k P_\nu U_k^\dagger =: p_\Delta \sum_{k:I_k\subset I}\underbrace{\frac{p_k}{p_\Delta}}_{=:q_k}\tilde{\omega}_{\delta_k} + (1-p_\Delta)\rho_{\mathrm{tail}} \tag{67}$$

with

$$p_\Delta = \mathrm{tr}\left(\Pi_\Delta \rho_\infty^{UHU^\dagger}\right), \tag{68}$$

$$p_k = \sum_{\nu\in W_k}\mathrm{tr}\left(\rho U_k P_\nu U_k^\dagger\right) = \mathrm{tr}\,(\rho\Pi_k), \tag{69}$$

$$\tilde{\omega}_{\delta_k} = \frac{1}{p_k}\sum_{\nu\in W_k} U_kP_\nu U_k^\dagger\rho_k U_k P_\nu U_k^\dagger, \tag{70}$$

$$\rho_{\mathrm{tail}} = \frac{1}{1-p_\Delta}\sum_{k:I_k\not\subset I}\sum_{\nu\in W_k} U_kP_\nu U_k^\dagger\rho_k U_k P_\nu U_k^\dagger. \tag{71}$$

To proceed, we start by bounding the entropy. We have

$$S(\tilde{\omega}_{\delta_k}) \geq S_2(\tilde{\omega}_{\delta_k}) = -\log\left(\mathrm{tr}\left(\tilde{\omega}_{\delta_k}^2\right)\right) \tag{72}$$

and

$$\mathrm{tr}\left(\tilde{\omega}_{\delta_k}^2\right) = \frac{1}{p_k^2}\sum_{\nu\in W_k}\mathrm{tr}\left((U_kP_\nu U_k^\dagger\rho_k)^2\right) = \frac{1}{p_k^2}\sum_{\nu\in W_k}\mathrm{tr}\left(U_k^{\otimes 2}P_\nu^{\otimes 2}U_k^{\otimes 2\dagger}\rho^{\otimes 2}\mathbb{F}\right) \tag{73}$$

where $\mathbb{F} = R_{(1,2)}$ flips the two tensor spaces. Then using the Weingarten formula as outlined in Section II yields

$$\begin{aligned}\mathbb{E}_{U\sim\mathcal{E}(\delta)}\left(\mathrm{tr}\left(\tilde{\omega}_{\delta_k}^2\right)\right) &= \frac{1}{p_k^2}\sum_{\nu\in W_k}\frac{\mathrm{tr}\,(P_\nu)}{d_k^2-1}\left[\mathrm{tr}\left((\rho_k)^2\right)\left(\mathrm{tr}\,(P_\nu) - \frac{1}{d_k}\right) + \mathrm{tr}\,(\rho_k)^2\left(1-\frac{\mathrm{tr}\,(P_\nu)}{d_k}\right)\right]\\ &\leq \frac{\mathrm{tr}\,(\rho_k)^2}{p_k^2}\sum_{\nu\in W_k}\frac{\mathrm{tr}\,(P_\nu)\,(1+\mathrm{tr}\,(P_\nu))}{d_k(d_k+1)} \leq \frac{1}{d_k+1}(1+\max{}_\nu \mathrm{tr}\,(P_\nu)) =: \frac{D_k+1}{d_k+1},\end{aligned} \tag{74}$$

where D_k is the degeneracy of the maximally degenerate eigenvalue inside W_k. By Markov's inequality

$$\Pr_{U\sim\mathcal{E}(\delta)}\left[\log(d_k) - S(\tilde{\omega}_{\delta_k}) \geq r\right] \leq \Pr_{U\sim\mathcal{E}(\delta)}\left[\operatorname{tr}\left(\tilde{\omega}^2_{\delta_k}\right) \geq \frac{1}{d_k}2^r\right] \leq \frac{(D_k+1)d_k}{d_k+1}2^{-r} \leq (D_k+1)2^{-r}. \tag{75}$$

Noticing that $\log(d_k) = S(\omega_{\delta_k})$ yields the result. Finally, we need to bound p_Δ. Notice that

$$p_\Delta = \operatorname{tr}\left(\Pi_\Delta \rho_\infty^{UHU^\dagger}\right) = \sum_{k:I_k\subset I}\sum_{\nu\in W_k} \operatorname{tr}\left(\rho U P_\nu U^\dagger\right) = \operatorname{tr}\left(\Pi_\Delta \rho\right). \tag{76}$$

We now wish to bound p_Δ using Theorem 3. By construction if $E_\nu \notin I$, then

$$|\operatorname{tr}(\rho H) - E_\nu| \geq \Delta \geq \omega(\sqrt{N}). \tag{77}$$

Then, by Theorem 3, we have

$$p_\Delta \geq 1 - \mathrm{e}^{-g_0\frac{\Delta^2}{N}} \tag{78}$$

if ρ is a product state and

$$p_\Delta \geq 1 - \mathrm{e}^{-g_\xi\left(\frac{\Delta^2}{N}\right)^{\frac{1}{D+1}}} \tag{79}$$

if ρ has exponential decay of correlations. □

Now the following version of Theorem 2 in the main text follows from Theorem 2 and Lemma 4.

Theorem 4 (Typical thermalization)**.** *Let H be a k-local Hamiltonian and ρ be a product state. Let $g_\beta(H)$ be the Gibbs state of H at inverse temperature β such that $|\operatorname{tr}(g_\beta(H)H) - \operatorname{tr}(\rho H)| \leq \sigma$. Assume $g_\beta(H)$ has exponential decay of correlations and $\sigma\zeta_N \leq R\ln(N)^{2D}N^{-\kappa}$ for $B,\kappa \geq 0$ constants. For any constant $\alpha \in [0,1)$, if $\delta = 3\sqrt{2\pi}N^{\frac{1-\alpha}{D+1}-\kappa}$, then with probability at least $1-(\mathcal{D}+1)\exp(-c_2 N^{\frac{1-\alpha}{D+1}})$ drawing U at random from $\mathcal{E}(\delta)$, we have*

$$D_l(\rho_\infty^{UHU^\dagger}, g_\beta(H)) \leq C_2\, N^{-\gamma_2\alpha} + N^{-\gamma_3(1-\alpha)}\ln(N)^{2D}, \tag{80}$$

where $c_2, C_2, \gamma_2, \gamma_3$ are system-size independent constants.

Proof. Let Δ such that

$$\mathrm{e}^{\Delta^2/\sigma^2} = \frac{N^{\frac{1-\alpha}{D+1}}}{R\ln(N)^{2D}}. \tag{81}$$

By applying Lemma 4 with $r := N^{\frac{1-\alpha}{D+1}}$ we have that

$$\rho_\infty^{UHU^\dagger} = p_\Delta \tau + (1-p_\Delta)\rho_{\text{tail}} \tag{82}$$

is approximately GmE satisfying the conditions of Theorem 2, hence we have

$$D_l(\rho_\infty^{UHU^\dagger}, g_\beta(H)) \leq C_2\, N^{-\gamma_2\alpha} + 2(1-p_\Delta) \leq C_2\, N^{-\gamma_2\alpha} + N^{-\gamma_3(1-\alpha)}\ln(N)^{2D}. \tag{83}$$

□

A similar theorem is immediate for states with exponential decay of correlations, but in this case

$$1 - p_\Delta \leq \exp\left(-\Omega\left(\ln\left(\frac{N^{\frac{1-\alpha}{D+1}}}{\ln(N)^{2D}}\right)\right)^{\frac{1}{D+1}}\right). \tag{84}$$

Equilibration also immediately follows from Lemma 4, as a matter of fact for any observable A in the case of non-degenerate energy gaps, that is if $E_\mu - E_\nu = E_{\mu'} - E_{\nu'}$ implies $(\mu,\nu) = (\mu',\nu')$. We have [6]

$$\begin{aligned}\Delta A_\infty &:= \lim_{T\to\infty}\frac{1}{T}\int_0^T \mathrm{d}t\,\operatorname{tr}\left(A(\rho^{UHU^\dagger}(t) - \rho_\infty^{UHU^\dagger})\right)^2 \leq \|A\|_\infty^2 \operatorname{tr}\left(\left(\rho_\infty^{UHU^\dagger}\right)^2\right) \leq \|A\|_\infty^2\left((1-p_\Delta)^2 + \sum_{k:I_k\subset I} q_k^2 \operatorname{tr}\left(\tilde{\omega}^2_{\delta_k}\right)\right) \\ &\leq \|A\|_\infty^2\left((1-p_\Delta)^2 + 2^r\sum_{k:I_k\subset I} q_k^2\frac{1}{d_k}\right) \leq \|A\|_\infty^2\left((1-p_\Delta)^2 + 2^r \max{}_k \frac{1}{d_k}\right).\end{aligned} \tag{85}$$

The condition of non-degenerate gaps is expected to hold generically for interacting systems. Then for example taking $r = \min_k \log(\sqrt{d_k})$ we have that with probability at least

$$1 - (\mathcal{D}+1)\max_k \frac{1}{\sqrt{d_k}}, \tag{86}$$

$\Delta A_\infty \le \|A\|_\infty^2 \Big((1-p_\Delta)^2 + 2^r \max_k \frac{1}{\sqrt{d_k}} \Big)$. Since d_k is expected to be exponentially large, the system equilibrates. In addition, we have the following statement.

Theorem 5 (Short-time evolution)**.** *For any state ρ and $U \sim \mathcal{E}(\delta)$ and any $t \ge 0$*

$$\|\mathrm{e}^{-iHt}\rho \mathrm{e}^{iHt} - \mathrm{e}^{-iUHU^\dagger t}\rho \mathrm{e}^{iUHU^\dagger t}\|_1 \le 2t\delta. \tag{87}$$

Proof. Let $V = \mathrm{e}^{-iHt}$ and $\tilde{V} = \mathrm{e}^{-iUHU^\dagger t}$. Then

$$\|V\rho V^\dagger - \tilde{V}\rho\tilde{V}^\dagger\|_1 = \|\tilde{V}^\dagger V\rho V^\dagger \tilde{V} - \rho\|_1 = \|\tilde{V}^\dagger V[\rho, V^\dagger\tilde{V}]\|_1 = \|[\rho, V^\dagger\tilde{V} - \mathbb{1}]\|_1 \le 2\|\rho\|_1\|V - \tilde{V}\|_\infty \tag{88}$$

where we have used Hölder's inequality and standard properties of the trace norm. Let $X = UHU^\dagger - H$, then

$$\|V - \tilde{V}\|_\infty = \|\mathrm{e}^{iHt} - \mathrm{e}^{iHt+iXt}\|_\infty. \tag{89}$$

Now let the function f be $f(s) = \mathrm{e}^{iHt+iXts}$, then

$$\|V - \tilde{V}\|_\infty = \|f(1) - f(0)\|_\infty \le \int_0^1 \mathrm{d}s\|f'(s)\|_\infty. \tag{90}$$

Then, using

$$f'(s) = it\int_0^1 \mathrm{d}r\, \mathrm{e}^{r(iHt+iXts)}X\mathrm{e}^{(1-r)(iHt+iXts)}, \tag{91}$$

we get

$$\|f'(s)\|_\infty \le t\|X\|_\infty \tag{92}$$

and hence by Lemma 3

$$\|V - \tilde{V}\|_\infty \le t\|X\|_\infty = t\|H - UHU^\dagger\|_\infty \le t\delta, \tag{93}$$

together with $\|\rho\|_1 = 1$ this proves the result. □

Choosing

$$\delta = \Omega(N^{\frac{1-\alpha}{D+1}-\kappa}), \tag{94}$$

if the Berry-Esseen error is bounded by $\tilde{O}(N^{-1/2-\kappa})$, we have, again choosing $\alpha \ge 1-\kappa(D+1)$, that δ is a decreasing function of the system size, and the two dynamics are indistinguishable up to ϵ until a time $\sim \epsilon N^{\kappa - \frac{1-\alpha}{D+1}}$. We now move on to proving that the Gibbs states of H and $UHU^\dagger$ are locally indistinguishable.

Theorem 6 (Local indistinguishability of perturbed Gibbs states)**.** *Let H be a k-local Hamiltonian. Let $g_\beta(H)$ be the Gibbs state of H at an inverse temperature β such that $g_\beta(H)$ has exponential decay of correlations. For any U drawn from $\mathcal{E}(\delta)$ and l, κ, α as in Theorem 2 we have*

$$D_l(g_\beta(H), g_\beta(UHU^\dagger)) \le C_3\, N^{-\gamma_4\alpha - \gamma_5\kappa}, \tag{95}$$

for system-size independent constants C_3, γ_4, γ_5.

Proof. A simple computation reveals

$$S(g_\beta(UHU^\dagger)\|g_\beta(H)) = \beta\mathrm{tr}\left((UHU^\dagger - H)g_\beta(H)\right) \le \beta\|H - UHU^\dagger\|_\infty \le \beta\delta \tag{96}$$

where we have used Lemma 3. The result follows from Corollary 1, with $\epsilon = \beta\delta N^{-\frac{1}{D+1}} = \beta N^{-\frac{\alpha}{D+1}-\kappa}$, which ends the proof. □

IV. SUPPLEMENTARY NOTE 4: RELAXATION DYNAMICS

We previously discussed the dynamics in the short-time regime, we now turn to discussing the typical late-time relaxation dynamics to the thermal state following an non-equilibrium initial preparation. We abbreviate in the following $\rho_U(t) := \mathrm{e}^{-iUHU^\dagger t}\rho \mathrm{e}^{iUHU^\dagger t}$. To prove the statement in the main text, we write

$$\langle A\rangle_{\rho_U(t)} - \langle A\rangle_{\rho_\infty^{UHU^\dagger}} = \langle A\rangle_{\rho_U(t)} - \mathbb{E}\left(\langle A\rangle_{\rho_U(t)}\right) + \mathbb{E}\left(\langle A\rangle_{\rho_U(t)}\right) - \mathbb{E}\left(\langle A\rangle_{\rho_\infty^{UHU^\dagger}}\right) + \mathbb{E}\left(\langle A\rangle_{\rho_\infty^{UHU^\dagger}}\right) - \langle A\rangle_{\rho_\infty^{UHU^\dagger}} \tag{97}$$

where the expectation values are taken over $U \sim \mathcal{E}(\delta)$. Hence

$$\left|\langle A\rangle_{\rho_U(t)} - \langle A\rangle_{\rho_\infty^{UHU^\dagger}}\right| \le \left|\langle A\rangle_{\rho_U(t)} - \mathbb{E}\left(\langle A\rangle_{\rho_U(t)}\right)\right| + \left|\langle A\rangle_{\rho_\infty^{UHU^\dagger}} - \mathbb{E}\left(\langle A\rangle_{\rho_\infty^{UHU^\dagger}}\right)\right| + R(t), \tag{98}$$

where we have defined the function R to be

$$R(t) := \left|\mathbb{E}\left(\langle A\rangle_{\rho_U(t)}\right) - \mathbb{E}\left(\langle A\rangle_{\rho_\infty^{UHU^\dagger}}\right)\right|. \tag{99}$$

We now need to show that the first two terms are small with high probability, and the promised decay of $R(t)$ with the relevant spectral assumptions.

In order to show that the first two terms are small with high probability, we will need to bound their second moments. From now on, let Π_k be the orthogonal projector onto the k-th window $\mathcal{W}_k$, and $d_k = \mathrm{tr}\left(\Pi_k\right)$ the number of energy eigenstates contained within. We denote as K the total number of windows. In addition, for any operator X, we define

$$X_{k,j} := \Pi_k X \Pi_j \qquad X_k = X_{k,k}. \tag{100}$$

We also define

$$\phi_k(t) := \frac{1}{d_k}\mathrm{tr}\left(\Pi_k \mathrm{e}^{-iHt}\right). \tag{101}$$

We now state the second moment bounds, which are proven in Section IV A under the assumption that H has no degenerate eigenstates. If H has weak degeneracies we expect very similar results to hold.

Lemma 5 (Closeness in expectation)**.**

$$\mathbb{E}\left(\left|\langle A\rangle_{\rho_U(t)} - \mathbb{E}\left(\langle A\rangle_{\rho_U(t)}\right)\right|^2\right) \le \|A\|_\infty^2 O\left(K^2 \sum_k \mathrm{tr}\left(\rho_k\right)\frac{1}{d_k}\right). \tag{102}$$

Lemma 6 (Closeness for infinite times)**.**

$$\mathbb{E}\left(\left|\langle A\rangle_{\rho_\infty^{UHU^\dagger}} - \mathbb{E}\left(\langle A\rangle_{\rho_\infty^{UHU^\dagger}}\right)\right|^2\right) \le \|A\|_\infty^2 O\left(\sum_k \mathrm{tr}\left(\rho_k\right)\frac{1}{d_k}\right). \tag{103}$$

These bounds imply the following:

Lemma 7 (Time-evolution concentration)**.** *For a Hamiltonian H, an observable A, and some initial state ρ it holds that*

$$\mathrm{Pr}_{U\sim\mathcal{E}(\delta)}\left[\left|\langle A\rangle_{\rho_U(t)} - \mathbb{E}\left(\langle A\rangle_{\rho_U(t)}\right)\right| + \left|\langle A\rangle_{\rho_\infty^{UHU^\dagger}} - \mathbb{E}\left(\langle A\rangle_{\rho_\infty^{UHU^\dagger}}\right)\right| \ge \epsilon\right] \le \|A\|_\infty^2 \frac{1}{\epsilon^2} O\left(K^2 \sum_k \mathrm{tr}\left(\rho_k\right)\frac{1}{d_k}\right). \tag{104}$$

$\sum_k \mathrm{tr}\left(\rho_k\right)\frac{1}{d_k}$ is the average number of eigenstates in the windows where ρ_k has a non negligible support. Since these windows are all in the bulk of the spectrum, it is expected that $\sum_k \mathrm{tr}\left(\rho_k\right)\frac{1}{d_k} \le \mathrm{e}^{-\Omega(N)}$.

Proof. Via a union bound, we get

$$\begin{aligned}
&\mathrm{Pr}_{U\sim\mathcal{E}(\delta)}\left[\left|\langle A\rangle_{\rho_U(t)} - \mathbb{E}\left(\langle A\rangle_{\rho_U(t)}\right)\right| + \left|\langle A\rangle_{\rho_\infty^{UHU^\dagger}} - \mathbb{E}\left(\langle A\rangle_{\rho_\infty^{UHU^\dagger}}\right)\right| \ge \epsilon\right] \\
&\quad \le \mathrm{Pr}_{U\sim\mathcal{E}(\delta)}\left[\left|\langle A\rangle_{\rho_\infty^{UHU^\dagger}} - \mathbb{E}\left(\langle A\rangle_{\rho_\infty^{UHU^\dagger}}\right)\right| \ge \epsilon/2\right] + \mathrm{Pr}_{U\sim\mathcal{E}(\delta)}\left[\left|\langle A\rangle_{\rho_U(t)} - \mathbb{E}\left(\langle A\rangle_{\rho_U(t)}\right)\right| \ge \epsilon/2\right] \\
&\quad \le \mathbb{E}\left(\left|\langle A\rangle_{\rho_U(t)} - \mathbb{E}\left(\langle A\rangle_{\rho_U(t)}\right)\right|^2\right)\frac{4}{\epsilon^2} + \mathbb{E}\left(\left|\langle A\rangle_{\rho_\infty^{UHU^\dagger}} - \mathbb{E}\left(\langle A\rangle_{\rho_\infty^{UHU^\dagger}}\right)\right|^2\right)\frac{4}{\epsilon^2} \le \|A\|_\infty^2 \frac{1}{\epsilon^2} O\left(K^2 \sum_k \mathrm{tr}\left(\rho_k\right)\frac{1}{d_k}\right).
\end{aligned} \tag{105}$$

□

We now move on to bounding $R(t)$. We have

$$\langle A\rangle_{\rho_U(t)} = \sum_{k,j} \mathrm{tr}\left(A_{j,k}U_k \mathrm{e}^{-iH_k t}U_k^\dagger \rho_{k,j} U_j \mathrm{e}^{iH_j t}U_j^\dagger\right) \tag{106}$$

and

$$\langle A\rangle_{\rho_\infty^{UHU^\dagger}} = \sum_k \sum_{\nu\in W_k} \mathrm{tr}\left(P_\nu U_k^\dagger \rho_k U_k P_\nu U_k^\dagger A U_k\right) \tag{107}$$

where W_k is the set of all ν such that $E_\nu \in I_k$. Invoking again the Weingarten formula as outlined in Section II, simple computations reveal for the expectation value

$$\mathbb{E}\left(\langle A\rangle_{\rho_U(t)}\right) = \sum_{k\neq j} \phi_k(t)\phi_j^*(t)\mathrm{tr}\left(A_{j,k}\rho_{k,j}\right) + \sum_k \frac{|\phi_k(t)|^2 d_k^2 - 1}{d_k^2-1}\mathrm{tr}\left(A_k\rho_k\right) + \frac{d_k}{d_k^2-1}(1-|\phi_k(t)|^2)\mathrm{tr}\left(A_k\right)\mathrm{tr}\left(\rho_k\right), \tag{108}$$

as well as

$$\mathbb{E}\left(\langle A\rangle_{\rho_\infty^{UHU^\dagger}}\right) = \sum_k \frac{|\overline{\phi}_k|^2 d_k^2 - 1}{d_k^2-1}\mathrm{tr}\left(A_k\rho_k\right) + \frac{d_k}{d_k^2-1}(1-|\overline{\phi}_k|^2)\mathrm{tr}\left(A_k\right)\mathrm{tr}\left(\rho_k\right). \tag{109}$$

Here, we have defined

$$|\overline{\phi}_k|^2 := \lim_{T\to\infty}\frac{1}{T}\int_0^T |\phi_k(t)|^2 \mathrm{d}t = \frac{1}{d_k^2}\sum_{\nu\in W_k} \mathrm{tr}\left(P_\nu\right)^2. \tag{110}$$

Then

$$\begin{aligned}\mathbb{E}\left(\langle A\rangle_{\rho_U(t)}\right) - \mathbb{E}\left(\langle A\rangle_{\rho_\infty^{UHU^\dagger}}\right) &= \sum_{k\neq j}\phi_k(t)\phi_j^*(t)\mathrm{tr}\left(A_{j,k}\rho_{k,j}\right) + \\ \sum_k \frac{1}{d_k+1}&\left[\mathrm{tr}\left(A_k\rho_k\right)\left(d_k^2\frac{|\phi_k(t)|^2 - |\overline{\phi}_k|^2}{d_k-1}\right) + \mathrm{tr}\left(\rho_k\right)\mathrm{tr}\left(A_k\right)\left(d_k\frac{|\overline{\phi}_k|^2 - |\phi_k(t)|^2}{d_k-1}\right)\right].\end{aligned} \tag{111}$$

Simplifying this expression, we get

$$\mathbb{E}\left(\langle A\rangle_{\rho_U(t)}\right) - \mathbb{E}\left(\langle A\rangle_{\rho_\infty^{UHU^\dagger}}\right) = \sum_{k\neq j}\phi_k(t)\phi_j^*(t)\mathrm{tr}\left(A_{j,k}\rho_{k,j}\right) + \sum_k \frac{d_k}{d_k+1}F_k(t)\left(\mathrm{tr}\left(A_k\rho_k\right) - \frac{\mathrm{tr}\left(A_k\right)}{d_k}\mathrm{tr}\left(\rho_k\right)\right) \tag{112}$$

where we have defined

$$F_k(t) := d_k\frac{|\phi_k(t)|^2 - |\overline{\phi}_k|^2}{d_k-1}. \tag{113}$$

This gives

$$R(t) \leq \|A\|_\infty K^2 \max{}_{k,j}\phi_k(t)\phi_j^*(t) + \max{}_k F_k(t)\sum_k\left|\mathrm{tr}\left(A_k\rho_k\right) - \frac{\mathrm{tr}\left(A_k\right)}{d_k}\mathrm{tr}\left(\rho_k\right)\right| \tag{114}$$

where we have used

$$\mathrm{tr}\left(A_{j,k}\rho_{k,j}\right) = \mathrm{tr}\left(A\Pi_k\rho\Pi_j\right) \leq \|A\|_\infty\|\Pi_k\rho\Pi_j\|_1 \leq \|A\|_\infty. \tag{115}$$

By the same reasoning, we can conclude that

$$\mathrm{tr}\left(A_k\rho_k\right) \leq \|A\|_\infty\|\rho_k\|_1 \tag{116}$$

and furthermore $\mathrm{tr}\left(A_k\right)/d_k \leq \|A\|_\infty$. This allows us to bound

$$\sum_k\left|\mathrm{tr}\left(A_k\rho_k\right) - \frac{\mathrm{tr}\left(A_k\right)}{d_k}\mathrm{tr}\left(\rho_k\right)\right| \leq 2\|A\|_\infty. \tag{117}$$

Altogether, we can conclude that

$$R(t) \leq \|A\|_\infty \left(K^2 \max_{k,j} |\phi_k(t)\phi_j^*(t)| + 2\max_k F_k(t)\right). \tag{118}$$

To make more progress, we need to be able to compute and discuss the functions ϕ_k. Mathematically speaking, we make a mild spectral assumption expected to be valid in natural quantum many-body systems. We assume that the above characteristic functions decay appropriately.

Assumption 1 (Spectral assumption)**.** *For some $r > 0$ and $t \leq \mathrm{poly}(N)$, we have*

$$|\phi_k(t)| \leq \frac{1}{t^r}. \tag{119}$$

In this case,

$$R(t) \leq O\left(\|A\|_\infty \frac{K^2}{t^{2r}}\right) \leq O\left(\|A\|_\infty \frac{N^2}{\delta^2 t^{2r}}\right). \tag{120}$$

where we use $K \leq O(N/\delta)$. Such a behavior can be derived, for example, by assuming a physically plausible slowly varying density of states inside each energy window I_k: consider the function $E_k(x) : [0,1] \to \mathbb{R}$ defined such that for $x \in \left[\frac{\nu}{d_k}, \frac{\nu+1}{d_k}\right)$, $E_k(x) = E_\nu$. Then, we have

$$\phi_k(t) = \int_0^1 \mathrm{d}x \mathrm{e}^{itE_k(x)}. \tag{121}$$

Now, define a linear interpolation of E_k: for $x \in \left[\frac{\nu}{d_k}, \frac{\nu+1}{d_k}\right)$

$$\tilde{E}_k(x) = d_k \Delta_\nu x + (\nu+1)E_\nu - \nu E_{\nu+1}, \tag{122}$$

where $\Delta_\nu = E_{\nu+1} - E_\nu$. $\tilde{E}_k(x)$ is piece-wise linear and satisfies $|\tilde{E}_k(x) - E_k(x)| \leq \max_{\nu \in W_k} \Delta_\nu$. In particular, defining

$$\tilde{\phi}_k(t) = \int_0^1 \mathrm{d}x \mathrm{e}^{it\tilde{E}_k(x)}, \tag{123}$$

we have $|\phi_k(t) - \tilde{\phi}_k(t)| \leq \max_{\nu \in W_k} \Delta_\nu$. Notice that $\tilde{E}_k(x)$ is strictly increasing hence invertible, and its inverse is continuous and differentiable almost everywhere. By the change of variable $y = \tilde{E}_k(x)$ we get

$$\tilde{\phi}_k(t) = \int_{e_k}^{e_{k+1}} \mathrm{d}y \mathrm{e}^{ity} \rho_k(y), \tag{124}$$

where $\rho_k(y) := \frac{\mathrm{d}}{\mathrm{d}y} \tilde{E}_k^{-1}(y)$ is the density of states, and satisfies $\rho_k(y) = \frac{1}{d_k \Delta_\nu}$ for $y \in [E_\nu, E_{\nu+1}]$. We take once again a piecewise linear interpolation of $\rho_k(y)$: for $y \in [E_\nu, E_{\nu+1}]$

$$\tilde{\rho}_k(y) = \frac{y - E_\nu}{d_k \Delta_\nu} \left(\frac{1}{\Delta_{\nu+1}} - \frac{1}{\Delta_\nu}\right) + \frac{1}{d_k \Delta_\nu} \tag{125}$$

is piecewise linear and satisfies

$$|\rho_k(y) - \tilde{\rho}_k(y)| \leq \max_{\nu \in W_k} \frac{1}{d_k} \left|\frac{1}{\Delta_{\nu+1}} - \frac{1}{\Delta_\nu}\right|. \tag{126}$$

Hence, defining

$$\tilde{\tilde{\phi}}_k(t) = \int_{e_k}^{e_{k+1}} \mathrm{d}y \mathrm{e}^{ity} \tilde{\rho}_k(y), \tag{127}$$

we have $|\tilde{\tilde{\phi}}_k(t) - \tilde{\phi}_k(t)| \leq \max_{\nu \in W_k} \frac{1}{d_k} \left|\frac{1}{\Delta_{\nu+1}} - \frac{1}{\Delta_\nu}\right|$, and furthermore, integrating by parts gives

$$\tilde{\tilde{\phi}}_k(t) = \frac{1}{it} \int_{e_k}^{e_{k+1}} \mathrm{d}y \tilde{\rho}_k(y) \frac{\mathrm{d}}{\mathrm{d}y} \mathrm{e}^{ity} = \frac{1}{it} \left(\mathrm{e}^{ite_{k+1}} \tilde{\rho}_k(e_{k+1}) - \mathrm{e}^{ite_k} \tilde{\rho}_k(e_k)\right) + \frac{1}{it} \int_{e_k}^{e_{k+1}} \mathrm{d}y \tilde{\rho}_k'(y) \mathrm{e}^{ity}. \tag{128}$$

Then

$$|\tilde{\tilde{\phi}}_k(t)| \leq \frac{1}{t}\left|\left(\mathrm{e}^{ite_{k+1}}\tilde{\rho}_k(e_{k+1}) - \mathrm{e}^{ite_k}\tilde{\rho}_k(e_k)\right)\right| + \frac{1}{t}\left|\int_{e_k}^{e_{k+1}} \mathrm{d}y \tilde{\rho}_k'(y)\mathrm{e}^{ity}\right| \\ \leq \frac{1}{t}\left(2 + \max_{\nu\in W_k}\frac{1}{d_k}\left|\frac{1}{\Delta_{\nu+1}} - \frac{1}{\Delta_\nu}\right|\right). \tag{129}$$

Overall, we get

$$|\phi_k(t)| \leq \frac{1}{t} + O\left(\max_{\nu\in W_k}\Delta_\nu\right) + O\left(\max_{\nu\in W_k}\frac{1}{d_k}\left|\frac{1}{\Delta_{\nu+1}} - \frac{1}{\Delta_\nu}\right|\right). \tag{130}$$

This shows that our assumption holds with $r = 1$ on time scales such that $1/t$ is large with respect to the spectral gaps and the difference of nearby spectral gaps. This is also compatible with natural expectations of spectral gaps following a Wigner-Dyson distribution, which due to level repulsion favors an almost uniform distribution of the spectral gaps. Altogether, using $K \leq O(N/\delta)$, this proves the following:

Theorem 7 (Closeness of observations)**.** *Under Assumption 1, with probability at least* $\frac{C}{\epsilon^2}\sum_k \mathrm{tr}\left(\rho_k\right)\frac{1}{d_k}$ *for a constant* C*,*

$$\left|\langle A\rangle_{\rho_U(t)} - \langle A\rangle_{\rho_\infty^{UHU^\dagger}}\right| \leq \epsilon + O\left(\|A\|_\infty \frac{N^2}{\delta^2 t^{2r}}\right). \tag{131}$$

A. Second moments bounds: proofs of Lemmas 5 and 6

We now prove the second moment bounds:

Proof of Lemma 5. We have

$$\langle A\rangle_{\rho_U(t)} = \sum_{k,j}\mathrm{tr}\left(A_{j,k}U_k\mathrm{e}^{-iH_kt}U_k^\dagger\rho_{k,j}U_j\mathrm{e}^{iH_jt}U_j^\dagger\right). \tag{132}$$

Then, let

$$a_{k,j}(U) := \mathrm{tr}\left(A_{j,k}U_k\mathrm{e}^{-iHt}U_k^\dagger\rho_{k,j}U_j\mathrm{e}^{iHt}U_j^\dagger\right). \tag{133}$$

We have

$$\mathbb{E}\left(\left|\langle A\rangle_{\rho_U(t)} - \mathbb{E}\left(\langle A\rangle_{\rho_U(t)}\right)\right|^2\right) = \sum_{k,j,r,s}\mathbb{E}(a_{k,j}(U)\bar{a}_{rs}(U)) - \mathbb{E}(a_{k,j}(U))\mathbb{E}(\bar{a}_{rs}(U)) = \sum_{k,j,r,s}\mathrm{Cov}(a_{k,j}(U), a_{rs}(U)). \tag{134}$$

By the Cauchy-Schwarz inequality

$$\mathrm{Cov}(a_{k,j}(U), a_{rs}(U)) \leq \sqrt{\mathrm{Var}(a_{k,j}(U))\mathrm{Var}(a_{rs}(U))}, \tag{135}$$

hence

$$\sum_{k,j,r,s}\mathrm{Cov}(a_{k,j}(U), a_{rs}(U)) \leq \left(\sum_{k,j}\sqrt{\mathrm{Var}(a_{k,j}(U))}\right)^2 \leq K^2\sum_{k,j}\mathrm{Var}(a_{k,j}(U)) \tag{136}$$

where $K = \sum_k = \Theta(N/\delta)$ is the total number of windows. In the case $k = j$, we have

$$\mathrm{Var}(a_{k,j}(U)) = \mathbb{E}(|a_{kk}(U)|^2) - |\mathbb{E}(a_{kk}(U))|^2 \leq \|A\|_\infty \mathrm{tr}\left(\rho_k^2\right) O\left(\frac{1}{d_k}\right) \tag{137}$$

(see Ref. [7], Eq. (11)). If $k \neq j$, one has

$$\mathrm{Var}(a_{k,j}(U)) = \mathbb{E}(|a_{k,j}(U)|^2) - |\mathbb{E}(a_{k,j}(U))|^2. \tag{138}$$

A simple computation reveals

$$\mathbb{E}(a_{k,j}(U)) = \mathrm{tr}\left(A\Pi_k\rho\Pi_j\right)\phi_k(t)\bar{\phi}_j(t). \tag{139}$$

The second moment of $a_{k,j}(U)$ can be rewritten as

$$\mathbb{E}(|a_{k,j}(U)|^2) = \mathrm{tr}\left(A_{j,k}\otimes A_{k,j}U_k\otimes U_j\mathrm{e}^{-iHt}\otimes \mathrm{e}^{iHt}U_k^\dagger\otimes U_j^\dagger\rho_{k,j}\otimes\rho_{j,k}U_j\otimes U_k\mathrm{e}^{iHt}\otimes\mathrm{e}^{-iHt}U_j^\dagger\otimes U_k^\dagger\right). \tag{140}$$

Another tedious but straightforward computation using Weingarten calculus as outlined in Section II reveals (we omit the time-dependence notation on the $\phi_k(t)$)

$$\begin{aligned}
\mathbb{E}(|a_{k,j}(U)|^2) &= \frac{|\mathrm{tr}\left(A_{j,k}\rho_{k,j}\right)|^2}{(d_k-1)^2(d_j-1)^2}\left[|\phi_k|^2|\phi_j|^2d_k^2d_j^2 - d_k^2|\phi_k|^2 - d_j^2|\phi_j|^2+1\right]\\
&+\frac{\|A_{j,k}\rho_{k,j}\|_F^2}{(d_k-1)^2(d_j-1)^2}\left[d_k(1-|\phi_k|^2)(d_j^2|\phi_j|^2-1)+d_j(1-|\phi_j|^2)(d_k^2|\phi_k|^2-1)\right]\\
&+\frac{\|A_{j,k}\|_F^2\|\rho_{k,j}\|_F^2d_kd_j}{(d_k-1)^2(d_j-1)^2}(1-|\phi_k|^2)(1-|\phi_j|^2)\\
&= |\mathrm{tr}\left(A_{j,k}\rho_{k,j}\right)|^2\left[|\phi_k|^2|\phi_j^2| + O\left(\frac{1}{d_k^2}\right)+O\left(\frac{1}{d_j^2}\right)\right]\\
&+\|A_{j,k}\rho_{k,j}\|_F^2\left[O\left(\frac{1}{d_k}\right)+O\left(\frac{1}{d_j}\right)\right]+\|A_{j,k}\|_F^2\|\rho_{k,j}\|_F^2O\left(\frac{1}{d_kd_j}\right),
\end{aligned} \tag{141}$$

where $\|\cdot\|_F$ indicates the Frobenius norm. Hence

$$\begin{aligned}
\mathbb{E}(|a_{k,j}(U)|^2) - |\mathbb{E}(|a_{k,j}(U))|^2 &= |\mathrm{tr}\left(A_{j,k}\rho_{k,j}\right)|^2\left[O\left(\frac{1}{d_k^2}\right)+O\left(\frac{1}{d_j^2}\right)\right]\\
&+\|A_{j,k}\rho_{k,j}\|_F^2\left[O\left(\frac{1}{d_k}\right)+O\left(\frac{1}{d_j}\right)\right]+\|A_{j,k}\|_F^2\|\rho_{k,j}\|_F^2O\left(\frac{1}{d_kd_j}\right).
\end{aligned} \tag{142}$$

Using repeatedly that for matrices A and B, $\|AB\|_F\le\|A\|_\infty\|B\|_F$, we have

$$\|A_{j,k}\rho_{k,j}\|_F^2 = \|\Pi_jA\Pi_k\rho\Pi_j\|_F^2 \le \|A\|_\infty^2\|\rho_{k,j}\|_F^2. \tag{143}$$

Furthermore,

$$\|A_{j,k}\|_F^2 = \|\Pi_jA\Pi_k\|_F^2 \le \min(d_j,d_k)\|A\|_\infty^2, \tag{144}$$

where we have used that $\|\Pi_j\|_F^2 = d_j$ and similarly for Π_k. Finally, by the Cauchy-Schwarz inequality

$$|\mathrm{tr}\left(A_{j,k}\rho_{k,j}\right)|^2 \le \|A_{j,k}\|_F^2\|\|\rho_{k,j}\|_F^2 \le \min(d_j,d_k)\|A\|_\infty^2\|\rho_{k,j}\|_F^2. \tag{145}$$

Putting everything together we get

$$\mathrm{Var}(a_{k,j}(U)) = \mathbb{E}(|a_{k,j}(U)|^2) - |\mathbb{E}(|a_{k,j}(U))|^2 = \|A\|_\infty^2\|\rho_{j,k}\|_F^2\left[O\left(\frac{1}{d_k}\right)+O\left(\frac{1}{d_j}\right)\right]. \tag{146}$$

By submultiplicativity of the Frobenius norm, we have that

$$\|\rho_{j,k}\|_F^2 = \|\Pi_j\rho\Pi_k\|_F^2 = \|\Pi_j\rho^{1/2}\rho^{1/2}\Pi_k\|_F^2 \le \|\Pi_j\rho^{1/2}\|_F^2\|\rho^{1/2}\Pi_k\|_F^2 = \mathrm{tr}\left(\rho_k\right)\mathrm{tr}\left(\rho_j\right). \tag{147}$$

Going back to Eq. (136), we have that in both the $k=j$ and $k\neq j$ case, using $\mathrm{tr}\left(\rho_k^2\right)\le\mathrm{tr}\left(\rho_k\right)^2$ for the former case,

$$\mathrm{Var}(a_{k,j}(U)) \le K^2\|A\|_\infty^2\mathrm{tr}\left(\rho_k\right)\mathrm{tr}\left(\rho_j\right)\left[O\left(\frac{1}{d_k}\right)+O\left(\frac{1}{d_j}\right)\right], \tag{148}$$

which yields

$$\mathbb{E}\left(\left|\langle A\rangle_{\rho_U(t)} - \mathbb{E}\left(\langle A\rangle_{\rho_U(t)}\right)\right|^2\right) \le \|A\|_\infty^2 O\left(K^2\sum_k\mathrm{tr}\left(\rho_k\right)\frac{1}{d_k}\right). \tag{149}$$

□

Proof of Lemma 6. We have

$$\langle A\rangle_{\rho_\infty^{UHU^\dagger}} = \sum_k \sum_{\nu\in W_k} \mathrm{tr}\left(P_\nu U_k \rho_k U_k^\dagger P_\nu U_k A_k U_k^\dagger\right). \tag{150}$$

Define $b_k(U)$ as

$$b_k(U) := \sum_{\nu\in W_k} \mathrm{tr}\left(P_\nu U_k \rho_k U_k^\dagger P_\nu U_k A_k U_k^\dagger\right) \tag{151}$$

so that

$$\langle A\rangle_{\rho_\infty^{UHU^\dagger}} = \sum_k b_k(U). \tag{152}$$

The $b_k(U)$ are independent as each only depends on the block U_k, hence we have

$$\mathbb{E}\left(\left|\langle A\rangle_{\rho_\infty^{UHU^\dagger}} - \mathbb{E}\left(\langle A\rangle_{\rho_\infty^{UHU^\dagger}}\right)\right|^2\right) = \sum_k \mathbb{E}(b_k(U)^2) - \mathbb{E}(b_k(U))^2. \tag{153}$$

We have

$$\begin{aligned}\mathbb{E}(b_k(U)) &= \frac{|\overline{\phi}_k|^2 d_k^2 - 1}{d_k^2-1}\mathrm{tr}\left(A_k\rho_k\right) + \frac{d_k}{d_k^2-1}(1-|\overline{\phi}_k|^2)\mathrm{tr}\left(A_k\right)\mathrm{tr}\left(\rho_k\right)\\ &= \frac{d_k}{d_k^2-1}(1-|\overline{\phi}_k|^2)\mathrm{tr}\left(A_k\right)\mathrm{tr}\left(\rho_k\right) + \|A\|_\infty \mathrm{tr}\left(\rho_k\right) O\left(\frac{1}{d_k}\right),\end{aligned} \tag{154}$$

where we have defined

$$|\overline{\phi}_k|^2 := \lim_{T\to\infty}\frac{1}{T}\int_0^T |\phi_k(t)|^2 \mathrm{d}t = \frac{1}{d_k^2}\sum_{\nu\in W_k}\mathrm{tr}\left(P_\nu\right)^2 = O\left(\frac{1}{d_k}\right). \tag{155}$$

We move on to the second moment: using the Weingarten formula (see Section II)

$$\begin{aligned}\mathbb{E}(b_k(U)^2) &= \sum_{\nu,\mu\in W_k}\mathbb{E}\left(\mathrm{tr}\left(P_\nu^{\otimes 2}\otimes P_\mu^{\otimes 2} U^{\otimes 4}\rho_k\otimes A_k\otimes\rho_k\otimes A_k U^{\dagger\otimes 4} R_{(1,2)}R_{(3,4)}\right)\right)\\ &= \sum_{\mu,\nu}\sum_{\sigma,\tau\in S_4}\mathrm{Wg}(\sigma\tau^{-1},d_k)\mathrm{tr}\left(P_\nu^{\otimes 2}\otimes P_\mu^{\otimes 2} R_\sigma R_{(1,2)}R_{(3,4)}\right)\mathrm{tr}\left(R_\tau \rho_k\otimes A_k\otimes\rho_k\otimes A_k\right).\end{aligned} \tag{156}$$

To simplify matters, from now on we will assume that the eigenvalues are non-degenerate, that is, $D_\nu = \mathrm{tr}\left(P_\nu\right) = 1$. As long as D_ν is at most polynomially large, generalization to the degenerate case is easy but tedious. We denote by $S_2\times S_2$ the set of permutations in S_4 consisting of $(), (1,2), (3,4), ((1,2),(3,4))$, that is, the permutation group of the first two elements and of the second two elements taken separately. Then we have

$$\mathrm{tr}\left(P_\nu^{\otimes 2}\otimes P_\mu^{\otimes 2} R_\sigma R_{(1,2)}R_{(3,4)}\right) = \begin{cases}1 & \text{if } \sigma\in S_2,\times S_2\\ \delta_{\mu,\nu} & \text{otherwise.}\end{cases} \tag{157}$$

Then

$$\mathbb{E}(b_k(U)^2) = \sum_{\tau\in S_4}\mathrm{tr}\left(R_\tau\rho_k\otimes A_k\otimes\rho_k\otimes A_k\right)\left[d_k^2\sum_{\sigma\in S_2\otimes S_2}\mathrm{Wg}(\sigma\tau^{-1},d_k) + d_k\sum_{\sigma\notin S_2\otimes S_2}\mathrm{Wg}(\sigma\tau^{-1},d_k)\right]. \tag{158}$$

This sum contains many terms, but we are only interested in showing that the potentially $O(1)$ terms are compensated by the $O(1)$ term in $\mathbb{E}(b_k(U))^2$ (see Eq. (154)), and that all other terms decay at least as $1/d_k$. We have that $\mathrm{Wg}(\sigma\tau^{-1},d_k) = O(d_k^{-4})$ if $\sigma=\tau$, and $\mathrm{Wg}(\sigma\tau^{-1},d_k) = O(d_k^{-5})$ otherwise (see Table S2). This implies that

$$d_k^2\sum_{\sigma\in S_2\otimes S_2}\mathrm{Wg}(\sigma\tau^{-1},d_k) + d_k\sum_{\sigma\notin S_2\otimes S_2}\mathrm{Wg}(\sigma\tau^{-1},d_k) \le \begin{cases}O(d_k^{-3}) & \text{if } \tau\notin S_2\times S_2,\\ O(d_k^{-2}) & \text{if } \tau\in S_2\times S_2.\end{cases} \tag{159}$$

Together with the fact that

$$\mathrm{tr}\left(R_\tau \rho_k \otimes A_k \otimes \rho_k \otimes A_k\right) \leq O(d_k^2)\|A\|_\infty^2 \mathrm{tr}\left(\rho_k\right)^2 \tag{160}$$

we get

$$\sum_{\tau \notin S_2 \times S_2} \mathrm{tr}\left(R_\tau \rho_k \otimes A_k \otimes \rho_k \otimes A_k\right) \left[d_k^2 \sum_{\sigma \in S_2 \otimes S_2} \mathrm{Wg}(\sigma\tau^{-1}, d_k) + d_k \sum_{\sigma \notin S_2 \otimes S_2} \mathrm{Wg}(\sigma\tau^{-1}, d_k) \right] \leq \|A\|_\infty^2 \mathrm{tr}\left(\rho_k\right)^2 O(1/d_k). \tag{161}$$

Now consider the permutations $\tau \in S_2 \times S_2$. Unless $\tau = ()$ or $\tau = (1,3)$,

$$\mathrm{tr}\left(R_\tau \rho_k \otimes A_k \otimes \rho_k \otimes A_k\right) \leq O(d_k)\|A\|_\infty^2 \mathrm{tr}\left(\rho_k\right)^2 . \tag{162}$$

Since $(1,3) \notin S_2 \times S_2$, this yields

$$\sum_{\tau \neq ()} \mathrm{tr}\left(R_\tau \rho_k \otimes A_k \otimes \rho_k \otimes A_k\right) \left[d_k^2 \sum_{\sigma \in S_2 \otimes S_2} \mathrm{Wg}(\sigma\tau^{-1}, d_k) + d_k \sum_{\sigma \notin S_2 \otimes S_2} \mathrm{Wg}(\sigma\tau^{-1}, d_k) \right] \leq \|A\|_\infty^2 \mathrm{tr}\left(\rho_k\right)^2 O(1/d_k). \tag{163}$$

The only remaining term is the one with $\tau = ()$. We simply bound

$$d_k^2 \sum_{\sigma \in S_2 \otimes S_2} \mathrm{Wg}(\sigma, d_k) + d_k \sum_{\sigma \notin S_2 \otimes S_2} \mathrm{Wg}(\sigma, d_k) \leq d_k^2 \sum_{\sigma \in S_4} \mathrm{Wg}(\sigma, d_k) = \frac{d_k^2}{d_k(d_k+1)(d_k+2)(d_k+3)} \leq \frac{1}{(d_k+1)^2}. \tag{164}$$

Finally,

$$\mathbb{E}(b_k(U)^2) \leq \|A\|_\infty^2 \mathrm{tr}\left(\rho_k\right)^2 O\left(\frac{1}{d_k}\right) + \mathrm{tr}\left(A_k\right)^2 \mathrm{tr}\left(\rho_k\right)^2 \frac{1}{(d_k+1)^2}. \tag{165}$$

In the case of non-degenerate eigenvalues, Eq. (154) reduces to

$$\mathbb{E}(b_k(U)) = \frac{1}{d_k+1} \mathrm{tr}\left(A_k\right) \mathrm{tr}\left(\rho_k\right) + \|A\|_\infty \mathrm{tr}\left(\rho_k\right) O\left(\frac{1}{d_k}\right) \tag{166}$$

which shows

$$\mathbb{E}(b_k(U)^2) - \mathbb{E}(b_k(U))^2 \leq \|A\|_\infty^2 \mathrm{tr}\left(\rho_k\right)^2 O\left(\frac{1}{d_k}\right). \tag{167}$$

Then

$$\mathbb{E}\left(\left|\langle A\rangle_{\rho_\infty^{UHU^\dagger}} - \mathbb{E}\left(\langle A\rangle_{\rho_\infty^{UHU^\dagger}}\right)\right|^2\right) \leq \|A\|_\infty^2 \sum_k \mathrm{tr}\left(\rho_k\right)^2 O\left(\frac{1}{d_k}\right) \leq \|A\|_\infty^2 O\left(\sum_k \mathrm{tr}\left(\rho_k\right) \frac{1}{d_k}\right). \tag{168}$$

□

V. SUPPLEMENTARY NOTE 5: TRANSLATIONAL INVARIANCE

We will now turn to discuss notions of translational invariance. More generally, we will show that if the original Hamiltonian has a certain symmetry $T : [H, T] = 0$, then the equilibrium state $\rho_\infty^{UHU^\dagger}$ with high probability has the same symmetry in an approximate form. Applying this to the translation symmetry yields the claim in the main text. We will first prove the following. Since we will rely on Lemma 6, we assume non-degenerate eigenvalues, with again the caveat that we expect weak degeneracies not to make a meaningful difference.

Lemma 8 (Auxiliary lemma)**.** *Let A be an observable such that $\mathrm{tr}\left(\Pi_i A\right) = 0$ for all energy windows I_i. Let $\epsilon > 0$. Then with probability at least*

$$1 - \frac{\|A\|_\infty^2}{\epsilon^2} \sum_k \Omega\left(\frac{1}{d_k}\right) \mathrm{tr}\left(\rho_k\right), \tag{169}$$

for U drawn from $\mathcal{E}(\delta)$ we have

$$\left|\mathrm{tr}\left(\rho_\infty^{UHU^\dagger} A\right)\right| \leq \epsilon. \tag{170}$$

Proof. We have

$$\Pr\left(\operatorname{tr}\left(\rho_\infty^{UHU^\dagger}A\right)^2 \geq \epsilon^2\right) \leq \frac{1}{\epsilon^2}\mathbb{E}\left(\operatorname{tr}\left(\rho_\infty^{UHU^\dagger}A\right)^2\right). \tag{171}$$

To upper bound $\mathbb{E}\left(\operatorname{tr}\left(\rho_\infty^{UHU^\dagger}A\right)^2\right)$, we proceed exactly as in the proof of Lemma 6. There, we have shown that (see Eq. (165))

$$\mathbb{E}\left(\operatorname{tr}\left(\rho_\infty^{UHU^\dagger}A\right)^2\right) = \sum_k \mathbb{E}(b_k(U)^2) \tag{172}$$

with

$$\mathbb{E}(b_k(U)^2) \leq \|A\|_\infty^2 \operatorname{tr}(\rho_k)^2\, O\left(\frac{1}{d_k}\right) + \operatorname{tr}(A_k)^2 \operatorname{tr}(\rho_k)^2 \frac{1}{(d_k+1)^2}. \tag{173}$$

By assumption, in this case $\operatorname{tr}(A_k) = 0$, hence

$$\mathbb{E}\left(\operatorname{tr}\left(\rho_\infty^{UHU^\dagger}A\right)^2\right) \leq \|A\|_\infty^2 \sum_k O\left(\frac{1}{d_k}\right)\operatorname{tr}(\rho_k) \tag{174}$$

which implies the result. □

As commented earlier, d_k might be smaller at the edge of the spectrum, but it is still expected to be polynomial in the system size, and the tails at the edge of the spectrum may be cut if the state has exponential decay of correlations. Then, we find the following corollary.

Corollary 2 (Preservation of symmetries)**.** *Suppose the Hamiltonian has no degenerate eigenvalues and let T be a unitary which commutes with the Hamiltonian. Let A be an observable, let $\epsilon > 0$, then with probability at least*

$$1 - \frac{\|A\|_\infty^2}{\epsilon^2}\sum_k \Omega\left(\frac{1}{d_k}\right)\operatorname{tr}(\rho_k) \tag{175}$$

for U drawn from $\mathcal{E}(\delta)$ we have that

$$|\operatorname{tr}\left((A - TAT^\dagger)\rho_\infty^{UHU^\dagger}\right)| \leq \epsilon. \tag{176}$$

Proof. Since H and T commute, T commutes with the projectors onto the eigenspaces of H, and in particular with the projectors Π_i. We then have

$$\operatorname{tr}\left(\Pi_i TAT^\dagger\right) = \operatorname{tr}(\Pi_i A). \tag{177}$$

We conclude by applying Lemma 8 to the observable $TAT^\dagger - A$. □

As a corollary, we get the statement in eq. (17) in the main text. Let A be a local observable and call A_C the observable A acting on the hypercube $C \in \mathcal{C}_l$. We have for any $C' \in \mathcal{C}_l$

$$\begin{aligned}\left|\operatorname{tr}\left(A_{C'}(g_\beta(H) - \rho_\infty^{UHU^\dagger})\right)\right| &\leq \frac{1}{|\mathcal{C}_l|}\sum_{C\in\mathcal{C}_l}\left|\operatorname{tr}\left(A_C(g_\beta(H) - \rho_\infty^{UHU^\dagger})\right)\right| + \left|\operatorname{tr}\left((A_{C'} - A_C)(g_\beta(H) - \rho_\infty^{UHU^\dagger})\right)\right| \\ &\leq D_l(\rho_\infty^{UHU^\dagger}, g_\beta(H)) + \frac{1}{|\mathcal{C}_l|}\sum_{C\in\mathcal{C}_l}\left|\operatorname{tr}\left((A_{C'} - A_C)\rho_\infty^{UHU^\dagger}\right)\right|.\end{aligned} \tag{178}$$

where we have used that $g_\beta(H)$ is translation invariant. Since $\operatorname{tr}((A'_C - A_C)\Pi_k) = 0$ for all k, as H is translation invariant, by Lemma 8 we have that with high probability

$$\left|\operatorname{tr}\left((A_{C'} - A_C)\rho_\infty^{UHU^\dagger}\right)\right| \leq \epsilon. \tag{179}$$

To see that the probability is exponentially close to 1, assuming all windows in a sub-extensive neighborhood of $\operatorname{tr}(\rho H)$ contain exponentially many eigenvalues, we have

$$\frac{\|A\|_\infty^2}{\epsilon^2}\sum_k \Omega\left(\frac{1}{d_k}\right)\operatorname{tr}(\rho_k) \leq \mathrm{e}^{-\Omega(N)}. \tag{180}$$

SUPPLEMENTARY REFERENCES

[1] F. G. S. L. Brandao and M. Cramer, "Equivalence of statistical mechanical ensembles for non-critical quantum systems," (2015), arXiv:1502.03263.
[2] A. M. Alhambra, PRX Quantum **4**, 040201 (2023).
[3] A. A. Mele, "Introduction to Haar measure tools in quantum information: A beginner's tutorial," (2024), arXiv:2307.08956.
[4] M. Fukuda, R. König, and I. Nechita, J. Phys. A **52**, 425303 (2019).
[5] A. Anshu, New J. Phys. **18**, 083011.
[6] A. J. Short, New J. Phys. **13**, 053009 (2011).
[7] P. Reimann, Nature Comm. **7**, 10821 (2016).
